# Reactions to caregiving during an intervention targeting frailty in community living older people

**DOI:** 10.1186/1471-2318-12-66

**Published:** 2012-10-25

**Authors:** Christina Aggar, Susan Ronaldson, Ian D Cameron

**Affiliations:** 1 Sydney Nursing School, University of Sydney, 88 Mallet Street, Camperdown, NSW 2050, Australia; 2 Rehabilitation Studies Unit, Faculty of Medicine, University of Sydney, Ryde, 2112, Australia

**Keywords:** Carers, Frailty, Caregiving, Older persons, Assessment

## Abstract

**Background:**

The demands and consequences of caregiving are considerable. However, such outcomes are not commonly investigated in the evaluation of interventions targeting frailty. This study aims to explore family carers’ reactions to caregiving during an intervention targeting frailty in community living older people.

**Method:**

A study of carers (n=119) embedded in a 12 month randomised controlled intervention targeting frailty in people 70 years or older, compared to usual care. Reactions to caregiving were measured in the domains of health, finance, self-esteem, family support and daily schedule. Anxiety and depression levels were also evaluated. Carer outcomes were measured at baseline, 6 months and 12 months and at 3 months post frailty intervention.

**Results:**

Carers of frail older people in the intervention group showed a sustained improvement in health scores during the intervention targeting frailty, while health scores for carers of the frail older people in the control group, decreased and therefore their health worsened (F=2.956, p=0.034). The carers of the frail older people in the intervention group reported overall better health (F=5.303, p=0.023) and self-esteem (F=4.158, p=0.044), and co-resident carers reported higher self-esteem (F=4.088, p=0.046). Anxiety levels increased for carers in both intervention and control groups (F=2.819, p=0.04).

**Conclusion:**

The inclusion of carers in trials targeting frail older people may assist in the identification of at-risk carers and facilitate the provision of information and support that will assist them to continue providing care. Further research that explores the features of frailty interventions that impact on the caregiving experience is recommended.

**Trial registration:**

Australian New Zealand Clinical Trials Registry: ACTRN12608000565347

## Background

Frailty is currently a major subject of investigation in ageing research. Whilst there is no collectively agreed definition or measure of frailty, it is a general decline in several physiological systems and characterised by decreased functional ability and disability, causing vulnerability to adverse outcomes, including falls, hospitalisation, institutionalisation and death [1]. Frailty affects a person's independence by decreasing their ability to carry out activities of daily living [2] and therefore places a strain and responsibility on family members or friends who provide caregiving support [3].

A primary concern of aged care policy makers and researchers is to target frailty, and thereby decrease disability, improve functional status and ultimately delay entry into residential aged care facilities. As a result, interventions targeting frailty in community living older people (*to be referred to as* ‘*frailty interventions*’ *from this point*) are increasingly being implemented. The interventions generally involve referrals, medication and/or dietary changes or monitoring, home visits and exercise programs and are strongly influenced by the support of a carer, usually a family member [4,5]. However, their emphasis remains typically one of a patient centred approach with little research committed to understanding the carer’s experience of caregiving during the intervention [6].

Ferrucci and colleagues [7] suggest that we need to understand the method by which frailty interventions are effective or not for both the care recipient and their carer. Of the few randomised controlled frailty interventions that have included carers, the outcome measurement has been carer burden. The results for carer burden levels have varied; with improvement [8], worsening [9], no change [10] and a small effect experienced by co-residing carers [11]. To our knowledge, no randomised controlled intervention targeting frailty has examined carer experiences post intervention.

The responsibility placed on carers during frailty interventions can make caregiving a complex experience [4]. Carers are often relied upon to encourage adherence to frailty intervention strategies, with some carers expected to learn how to deal with complicated treatments, medications and provide symptom management [12,13]. Schulz [14] questions whether a desired effect for the older care recipient equates to a preferred carer effect. For example, a frailty intervention may involve attending regular group counselling sessions. If the carer is required to provide transport, this may be at the expense of the carer’s social or employment commitments. If as a consequence carer burden is experienced this may have a negative impact on the care recipient which may then influence the frailty intervention outcome. Alternatively, an intervention targeting frailty may have a positive impact on the carer. It has been suggested that the inclusion of carers in frailty intervention outcome assessments may produce a more positive or successful intervention for all involved [14,15]. Frailty levels and disability impact adversely on carers [16] and so the exclusion of carers from outcome assessment ultimately negates a broader perspective of who benefits from interventional research that targets frailty.

The potential physical, emotional and social impact of providing care [17] is further cause to include reactions to caregiving in frailty intervention evaluations. Carers can experience a significant decline in their own physical and mental health, as well as a negative impact on their employment and education prospects, their financial position, and their ability to participate in social and community life [17-19]. Carers also have significantly higher levels of depression and anxiety compared to the general population [18]. The most outstanding feature of caregiving research is the heterogeneity among reactions to caregiving. Reactions to caregiving are complex and diverse, and often relate to the carers’ positive or negative perception of their experience. Satisfying components of caregiving include improved relationships and strengthening of bonds, increased self-esteem and personal achievement in providing care [20].

The considerable consequences of reactions to caregiving necessitate the inclusion of carers in the investigation and evaluation of frailty interventions. The aim of this study is to examine carers’ reactions to caregiving during a frailty intervention for community living older people and three months post intervention.

## Method

### Participants

This is a longitudinal study of carers embedded in a 12 month randomised controlled trial targeting frailty in community living older people (≥70yrs). Carers nominated by the frail older person participating in a frailty intervention were invited to participate.

### Frailty intervention

The frailty intervention is a multicomponent interdisciplinary intervention, known as the ‘Frailty Intervention Trial’ (FIT). The intervention is a single centre randomised controlled trial targeting identified characteristics of frailty [21] in people aged 70 years and older and is compared to ‘usual care’ (general practitioner and community services). The FIT research protocol is published elsewhere [22]. Briefly, the frailty intervention inclusion criteria comprised participants: aged 70 years or older; recently discharged from an aged care or rehabilitation service; residing in metropolitan Sydney (Australia); not receiving rehabilitation services; without severe cognitive impairment; and expected to live more than 12 months. Frailty was empirically derived according to Fried and colleagues’ criteria [21]. Frailty exists when three or more of the following criteria were present: unintentional weight loss; fatigue; decreased grip strength; slow gait speed; or low physical activity. The aim of the trial is to examine the effects of the intervention on frailty, mobility, hospitalisation and institutionalisation in frail older people.

Case management facilitated and coordinated the delivery of the frailty intervention. The frailty intervention is tailored to each participant, based on their frailty characteristics and any problems detected following a geriatric evaluation at initial assessment. Components of the frailty intervention include: management of chronic health conditions, nutritional advice and supplements, a physiotherapy component, falls risk management, and provision of services to help at home. Ethical approval was sought from The Northern Sydney & Central Coast Health Human Research Ethics Committee and granted (Research Protocol Number 0709-191M).

### Recruitment

Permission from the participant enrolled in the frailty intervention to contact their nominated carer was requested at initial assessment and an information sheet outlining the study was made available for their carer to read. The research nurse responsible for recruitment into the frailty intervention used the following standard criteria when defining a carer: *a carer is a person who provides unpaid care to a family member or friend*. *This ranges from supervision to assistance with personal care*, *mobility and communication*, *through to emotional support and practical and financial assistance*[23].

Initial contact with the carer was via telephone from a researcher who was blinded to group allocation. Eligible carers indicating an interest in the study were sent the study questionnaire and an information sheet, along with a reply paid envelope. Information was obtained at baseline and follow-up outcome measures were collected at 6 months and 12 months and at 3 months post frailty intervention. At each time point the study participant carers were sent an information sheet indicating the follow-up period and the study questionnaire along with a reply paid envelope. Return of the questionnaire was implied consent by the carer.

### Inclusion and exclusion criteria

Eligible participants were the nominated family member or friend who assists or supervises the older frailty intervention participant with activities of daily living, such as personal care, mobility, communication, emotional and/or practical and financial support. In line with the randomised controlled trial’s criteria, carers whose care recipient became too ill, entered a nursing home or died during the FIT intervention, were required to withdraw from this study.

### Carer demographic measurements

The background questionnaire delivered at baseline determined carer and caregiving characteristics and formal support services utilised. Carer characteristics included age, gender, relationship to the patient, employment status and self-perceived health (excellent, very good, good, fair or poor). The caregiving characteristics included length of time in caregiving role, number of caregiving hours per week, co-residence and degree of care recipient frailty (very frail or frail). The receipt of formal support services (yes or no) was also recorded, including type of service.

### Outcome measurements

The Hospital Anxiety Depression Scale (HADS) [24] measured the emotional health of carers; it is clinically relevant and sensitive to changes over time [25]. The HADS has been rigorously psychometrically tested and is validated for use in the community with carers and across age groups [26]. The HADS is a 14 item, validated tool with two subscales: anxiety and depression. Each item is scored from 0 to 3; the maximum score is 21 for anxiety and depression. For inclusion of all possible cases, the lower end of the borderline score range was utilised. A score ≥8 indicates borderline levels of anxiety or depression. A score ≥11 indicates the presence of abnormal levels of anxiety or depression.

The Caregiver Reaction Assessment (CRA) [27] measured the caregiving experience. The CRA is a 24 item rigorously psychometrically tested multidimensional, 5-factor measure designed to assess the negative and positive aspects of the caregiving experience and changes in reaction over time, in an informal situation. The CRA measures: Daily schedule (5 items) – impact of providing care on the carer’s usual activities; Financial situation (3 items) – financial strain resulting from the caregiving situation; Health problems (4 items) – energy and physical capacity; Family support (5 items) – perceived family support or abandonment; Self-esteem (7 items) – self-worth, a positive experience as a result of caregiving. Responses are rated on a five point Likert scale, the format 1 = strongly disagree, 2 = disagree, 3 = neither agree nor disagree, 4 = agree, 5 = strongly agree. An unweighted mean score for each subscale and a single mean summary score is generated, ranging from 1.0 to 5.0. The reversal of the positive dimension (self-esteem) scoring, converts all dimensions of the scale with a higher score to indicate negative experiences. The CRA has been used in carer studies of persons with chronic, physical and mental conditions [27]. The HADS and the CRA (5 items) constituted the outcome measures, and were collected at baseline, 6 months and 12 months and with a follow-up measure at 3 months post intervention.

### Statistical analysis

At distribution and coding the researchers were unaware of the group to which the care recipients had been randomised. During follow-up data collection the carer’s group allocation was disclosed to the researchers. Whilst blinded at baseline allocation, due to the integral nature of the research design, this disclosure was unavoidable. Independent sample *t*-tests, chi-square and Pearson’s correlation were conducted to determine if initial participating carers and refusals differed with respect to gender, relationship and degree of frailty, and also to compare baseline outcome measures of the intervention and control groups.

Primary analysis explored reactions to caregiving between both intervention and control groups during the 12 month frailty intervention and 3 months post cessation of the frailty intervention. Secondary analysis explored whether key predictors of the HADS and CRA observed in previous cross sectional analyses determined the trajectories of caregiving reactions and carer well-being during the intervention [28,29].

The Linear Mixed Models Procedure (LMM) was used for both primary and secondary analyses. LMM is appropriate when analysing the same sample on repeated measures over time, to examine group differences in the sample on a particular measure and identify possible mediators of intervention effects. The complex nature and diversity of reactions to caregiving over several points in time make the LMM procedure particularly relevant due to the ability to integrate intercept and rate of change [30]. This procedure is also appropriate when data are missing at random, it allows variability in regression slopes and interdependence between the cases [31]. The Linear Mixed Model procedure within SPSS v19 was utilised, using restricted maximum likelihood. A number of models were explored and those with the lowest Akaike’s information criterion were used to assess the best fitting models, all of which used the first order autoregressive covariance structure, as measurements further apart in time were less correlated.

A statistically significant interaction between group and time in the primary analysis indicated that any change in reactions to caregiving over time is potentially influenced by the frailty intervention. For the primary and secondary analyses, group and time were entered as fixed factors, and their interaction was explored. For both primary and secondary analyses the intercept and individual subjects were included as random factors. As time was categorically coded, difference in means rather than changes in slope were explored. All four time measurements were included in the model. Survival analysis was used to explore loss to follow-up between the two groups. Utilising the SPSS program v19 *Missing Value Analysis*, Little’s MCAR test was conducted to determine if the data were missing completely at random. Sensitivity analysis of complete case data only was conducted to explore the impact of missing data.

### Sample size

Power analyses were conducted based on the linear mixed model procedures developed by Hedeker and colleagues [32] for 2-group repeated-measures designs in which a linear treatment by time interaction is expected. Previous studies found medium to strong correlations over time for CRA between .62-.83 [33,34], and for HADS between .63-.80 [25,26]. Streiner and Norman [35] found that, for a range of self-report survey tools, meaningful differences tended to average around 0.5 of a standard deviation (a medium effect size using Cohen’s d). In the absence of effect size guidelines, the medium effect size of 0.5 was adopted for the CRA and HADS. With a between-time correlation of .70 for both CRA and HADS, 34 participants per group were needed to detect an effect of 0.50 with a power of 0.80.

## Results

### Participant flow

**Figure 1:**
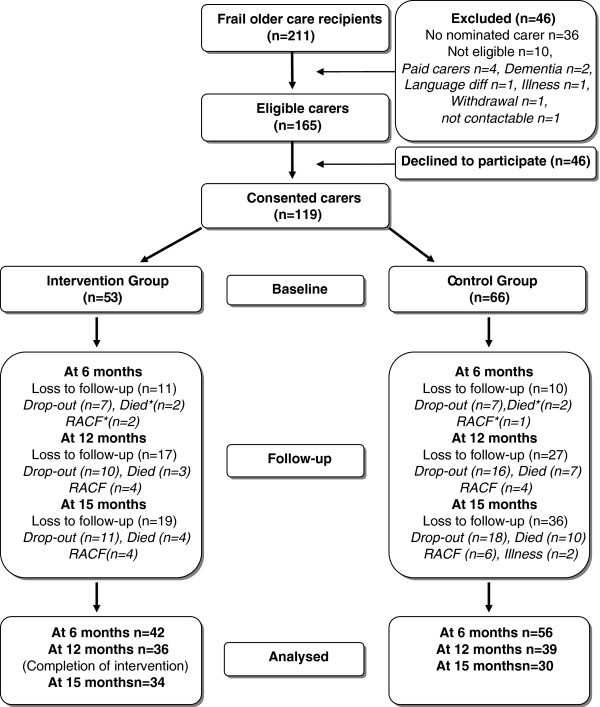
Study Flow Diagram.

Of the 241 FIT participants, 211 of them had carers potentially eligible to participate in this study. Ten frailty intervention participants were not eligible, and 36 did not identify or nominate a carer resulting in a total of 165 eligible carers of which 72% (n=119) gave informed consent and provided baseline data (Figure 1). No differences were found between participating carers and refusals with respect to gender, relationship and degree of frailty (this data was obtained by the research nurse during recruitment of older persons for the frailty intervention).

Reasons for and numbers of carers lost to follow-up are described in the Frailty Caregiver Study Flow Diagram (Figure 1). The final sample of carers (n=66) who completed questionnaires at all 4 data collection periods were compared to those lost to follow-up (n=55) from the baseline sample (n=119). Figure 1 illustrates that from the total number of carers lost to follow-up (n=55), 26 (49%) were required to withdraw as a result of the study’s exclusion protocol. Reasons included, care recipient’s death (n=14; 26% of total loss to follow-up), nursing home placement (n=10; 19% of the total loss to follow-up) and illness (n=2; 4% of total loss to follow-up). The loss to follow-up of these 26 carers (49%) was therefore due to factors related to FIT criteria of the care recipient rather than carer characteristics, and can therefore be considered to be at least missing at random (MAR). Carers who dropped out of the study for unknown reasons totalled 29 (51% of the total loss to follow-up).

Kaplan-Meier survival analysis demonstrated that the intervention and control groups were similar in their loss to follow-up and that there was no difference in mean length of time before becoming lost to follow-up. Mean time to ‘loss to follow-up’ was 12.96 months for the intervention group and 12.77 months for controls (p = 0.06). Cox Regression demonstrated that the relative risk of dropping out between the two groups was 1.6 (95% CI 0.926 – 2.8, p=0.09). For the 51% of carers who were lost to follow-up for reasons unknown, Little’s MCAR test in this study resulted in a chi-square of 0.935, (df = 21, p = 1.000) which indicates that the data are likely to be missing completely at random [36].

### Analysis of baseline data

No significant differences between carers of frail elderly in the intervention and control groups on demographic (Table 1) and baseline outcome variables were found and all measures were normally distributed (Table 2). The carers ranged in age from 37 to 94 years with a mean age (SD) of 66.68 (13.75) years. The majority were female (59.7%, n=71), and the relationship to the older person: 46.2% (n=55) were spouses, 37.8% (n=45) daughters and 16% (n=19) sons. Of the carers 57.1% (n=68) co-resided with their frail older relative. The majority of the carers were retired (56.3% n=67), 15.1% (n=18) were employed full-time and 22.7% (n=27) part-time, and 4.2% (n=5) were unemployed.

**Table 1:** Demographic details of intervention and control groups at baseline

**Variables**	**Intervention**	**Control**	***p *Value**
	**(n=53)**	**(n=66)**	
**Age, † yr**	64.98 (14.65)	68.05 (12.94)	0.229
**Gender ‡**			0.815
Female	31 (26.1)	40 (33.6)	
Male	22 (18.5)	26 (21.8)	
**Employment ‡**			0.270
Yes	24 (20.2)	21 (17.6)	
No	2 (1.7)	5 (4.2)	
Retired	27 (22.7)	40 (33.6)	
**Health Status ‡**			0.471
Excellent	7 (5.9)	6 (5.0)	
Very good	20 (16.8)	17 (14.3)	
Good	16 (13.4)	24 (20.2)	
Fair	9 (7.6)	18 (15.1)	
Poor	1 (0.8)	1 (0.8)	
**Relationship to Patient ‡**			0.573
Husband	13 (10.9)	15 (12.6)	
Wife	9 (7.6)	18 (15.1)	
Daughter	21 (17.6)	24 (20.2)	
Son	10 (8.4)	9 (7.6)	
**Co-resides ‡**			0.221
Yes	27 (22.7)	41 (34.5)	
No	26 (21.8)	25 (21)	
**Length of time caring †**	4.89 (3.64)	5.27 (5.16)	0.661
*95*% *CI*			
**Hours of care per week**			0.594
<20 hrs	31 (26.1)	33 (27.7)	
20-39 hrs	10 (8.4)	17 (14.3)	
40+ hrs	12 (10.1)	16 (13.4)	
**Formal Support‡**			0.774
Yes	34 (28.5)	44 (37)	
No	19 (16)	22 (18.5)	
**Level of Frailty‡**			0.910
Frail	34 (28.6)	43 (36.1)	
Very Frail	19 (16)	23 (19.3)	

† values given as the mean (SD), ‡ values given as No. (%).

**Table 2:** Depression, anxiety and reactions to caregiving at baseline

**Variable**	**Case group**	**Control group**	***t***	***df***	***p***
	**(n=53)**	**(n=66)**			
	**Mean (SD)**	**Mean (SD)**			
**HADS**					
Anxiety	6.4 (4.0)	6.2 (4.4)	0.20	116	0.84
Depression	5.2 (3.8)	4.8 (3.6)	0.47	116	0.64
**CRA**					
Schedule	3.1 (0.9)	2.3 (0.9)	0.38	117	0.70
Health	2.4 (0.6)	2.6 (0.8)	−1.01	117	0.32
Family support	2.4 (0.8)	2.3 (0.8)	0.62	117	0.54
Financial problem	2.3 (0.7)	2.5 (0.9)	−0.93	117	0.35
Self esteem	2.1 (0.6)	2.3 (0.6)	−1.64	117	0.10

Most of the care recipients had sustained needs with the mean duration of caregiving being 5.1 (4.51) years and approximately 20% of carers had provided care for more than 10 years. The most prevalent type of assistance provided by carers was transport (80%, n=95). At least 17% carers (n=20) were provided with an information pack specific to carers developed by the National Carers Association.

### Primary analysis

For the HADS, there was an effect of time for anxiety (F=2.819, p=0.04), with scores for carers of care recipients from both groups showing an increase (worsening anxiety level) over the duration of the study (Table 3). There were no statistically significant differences based on group membership or time, or interactions between group membership and time for depressive symptoms from the HADS.

**Table 3:** **Anxiety, depression and reactions to caregiving,**^**a**^**by group membership, at baseline, 6 months, 12 months and 15 months (3 months post intervention)**

	**Intervention group**		**Control group**	
	**mean (SE)**	**95% CI**	**mean (SE)**	**95% CI**
***HADS***				
**Anxiety**^**1**^				
Baseline	6.365 (0.571)	5.238-7.493	6.212 (0.506)	5.211-7.213
6 months	6.796 (0.598)	5.616-7.975	6.828 (0.527)	5.788-7.867
12 months	6.540 (0.616)	5.325-7.754	6.964 (0.565)	5.851-8.077
15 months	7.414 (0.622)	6.187-8.642	7.009 (0.598)	5.830-8.187
**Depression**				
Baseline	5.154 (0.517)	4.132-6.175	4.833 (0.459)	3.927-5.740
6 months	4.987 (0.534)	3.916-6.058	4.853 (0.478)	3.909-5.797
12 months	5.013 (0.565)	3.898-6.127	5.919 (0.520)	4.894-6.944
15 months	5.379 (0.575)	4.246-6.513	5.785 (0.560)	4.682-6.888
***CRA***				
**Health**^**2**^				
Baseline	2.439 (0.099)	2.244-2.633	2.576 (0.088)	2.401-2.750
6 months	2.393 (0.104)	2.188-2.599	2.654 (0.093)	2.471-2.838
12 months	2.259 (0.111)	2.041-2.477	2.764 (0.103)	2.561-2.967
15 months	2.586 (0.114)	2.361-2.810	2.795 (0.114)	2.571-3.019
**Schedule**				
Baseline	3.057 (0.119)	2.822-3.291	2.994 (0.107)	2.784-3.204
6 months	3.066 (0.125)	2.819-3.313	3.056 (0.112)	2.836-3.277
12 months	2.948 (0.132)	2.688-3.209	3.129 (0.123)	2.887-3.371
15 months	3.177 (0.135)	2.910-3.444	3.098 (0.134)	2.834-3.362
**Financial Problems**				
Baseline	2.333 (0.112)	2.111-2.555	2.475 (0.101)	2.276-2.674
6 months	2.329 (0.117)	2.097-2.561	2.513 (0.105)	2.306-2.720
12 months	2.316 (0.124)	2.072-2.560	2.649 (0.114)	2.423-2.874
15 months	2.467 (0.128)	2.215-2.718	2.648 (0.125)	2.401-2.895
**Family Support**				
Baseline	2.392 (0.114)	2.168-2.617	2.303 (0.102)	2.102-2.504
6 months	2.445 (0.118)	2.213-2.677	2.412 (0.105)	2.205-2.620
12 months	2.283 (0.123)	2.041-2.526	2.460 (0.113)	2.237-2.682
15 months	2.525 (0.126)	2.276-2.773	2.445 (0.122)	2.204-2.685
**Self esteem**^**3**^				
Baseline	2.137 (0.088)	1.963-2.312	2.327 (0.079)	2.170-2.483
6 months	2.095 (0.091)	1.916-2.275	2.365 (0.081)	2.204-2.525
12 months	2.112 (0.094)	1.926-2.297	2.384 (0.086)	2.215-2.554
15 months	2.154 (0.095)	1.966-2.341	2.353 (0.090)	2.175-2.532

^a^Marginal means based upon a linear mixed model with time and intervention group membership and their interaction as fixed factors. Random factors were intercepts and individual participants as fixed effects.

^1^Time p=0.04, Group/time and Group NS.

^2^Time p=0.043, Group/time p=0.034, Group p=0.023.

^3^Group p=0.044, Time and Group/time NS.

**Figure 2:**
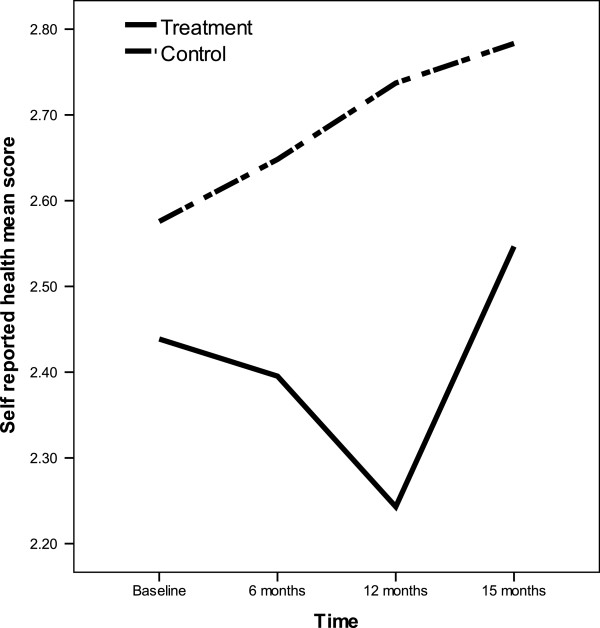
Significant treatment by time interaction on carer health (p= 0.034).

For the CRA, there were statistically significant differences based on group membership for ‘health’ (F=5.303, p=0.023) and ‘self-esteem’ (F=4.158, p=0.044), indicating that the carers of frail elderly in the intervention group reported overall better health and self-esteem (lower scores) than the carers of frail elderly in the control group. There was a statistically significant association for the CRA health based on time (F=2.787, p=0.043) and a statistically significant interaction between group membership and time for CRA health. The carers of frail elderly in the intervention group showed a sustained improvement in health (decrease in scores) while participating in the frailty intervention (F=2.956, p=0.034), with worsening (increasing scores) at 3 months post cessation of the frailty intervention (15 months, Figure 2). In contrast, carers of frail elderly in the control group showed a sustained worsening in health (increase in scores) over the entire duration of the study. For CRA items finance, family support and daily schedule, there were no statistically significant differences found for group membership or time, or interactions between group membership and time.

Sensitivity analysis of complete case data was conducted to explore the impact of missing data. Whilst coefficients differed, overall findings remained the same, with the only difference being an interaction between group membership and time for family support (p=0.04), indicating that, compared to carers of frail elderly in the control group, self-esteem scores for carers of frail elderly in the intervention group improved at 12 months.

### Secondary analysis

Predictor variables included in secondary analysis were self-perceived health status, relationship, hours of care per week, years spent caring, gender, co-residence and age. Each of these was assessed univariately against the outcomes and included in the LMM model if p < 0.10. Co-resident carers (that is, carers living with the elderly person) had statistically significant lower (better)scores for the CRA subscale ‘self-esteem’ (F=4.088, p=0.046), otherwise there were no statistically significant associations between outcomes and carer demographic characteristics.

Effect sizes between the intervention and control groups for all outcomes at all time periods were in general small, and ranged from 0.008 to 0.8 with only the effect size for health at 12 months and self-esteem at 6 months and 12 months being above the proposed clinically relevant value of 0.5 (effect size for health at baseline −0.2, 6 months −0.4, 12 months −0.8 and 15 months −0.3 and for self-esteem at baseline 0.3, 6 months 0.5, 12 months 0.5 & 15 months 0.4).

## Discussion

The aim of this study was to examine carers’ reactions to caregiving during a frailty intervention for community living older people. The intervention was individually tailored based on frailty characteristics and geriatric evaluation, and facilitated and coordinated via case management. The caregiving experience was explored in the domains; health, finance, self-esteem, family support and daily schedule, and anxiety and depression symptoms were measured. The carers of frail elderly in the intervention group reported overall better health and self-esteem than the carers of frail elderly in the control group. The carers of the frail older people receiving the intervention showed a sustained improvement in health scores whilst participating in the study, whereas the health scores of carers of the frail older people in the control group decreased. Anxiety levels for carers of the frail elderly in intervention and control groups increased. Secondary analysis demonstrated co-resident carers’ reported significantly higher self-esteem than those carers who did not reside with their frail older relative.

Carers of the frail elderly in the intervention group reported significantly better health and higher self-esteem than the carers of frail elderly in the control group. We propose this difference may be attributed to the case management feature of the intervention. The utilisation of a case management model approach in frailty research, particularly one that incorporates family orientated case management, has proven effective in improving frail older care recipients’ health, functional ability and satisfaction, and in reducing institutionalisation [37]. Whilst there is no specific evidence of the effectiveness of case management on carer health and general wellbeing or reducing carer burden or depression [38,39], research has found that barriers (e.g. preferences, resistance, lack of information) in obtaining health care services (e.g. transport) were associated with low carer self-rated health [40,41]. Home help, transport assistance, home modifications, assistive devices and respite were provided as part of the overall frailty intervention. The case management feature of the frailty intervention may have assisted in removing some of the barriers to the utilisation of these services, thus improving the carer’s self-reported health scores. Carer information, consultation and practical advice were also provided as part of the intervention. Self-esteem has been associated with the ability to manage the caregiving situation [20,42] and there is weak evidence that case management improves carer satisfaction by providing information support [38,39]. Carer self-esteem has positive implications for a carer’s well-being, and their ability to continue to provide care and support to community living frail older people. Further research involving carers in frailty interventions and the impact of case management models is recommended.

Whilst the case management model and the introduction to support services may explain the improvement of the carer’s self-reported health scores, it does not explain the subsequent decline in self-reported health scores three months cessation of the intervention. The additional supports were permanent services and continued post intervention unless terminated by the frail older person. However, it was not in the scope of this study to determine what components of the frailty intervention impacted on the caregiving experience. Further research that explores the features of frailty interventions that contribute to an improvement in carer self-rated health scores, including follow-up of post intervention is recommended.

Anxiety levels increased for carers of frail older people recruited to both the intervention and control groups. Anxiety is considered a normal reaction to a difficult situation. Generally, periods of anxiety are brief, however providing care to a frail older person can be a prolonged experience involving multiple stressful incidents, problems and challenges [43]. High levels of carer anxiety have been associated with a poor interpersonal relationship with the care recipient, ethnic and cultural issues, role conflict, reduced time for self and financial concerns; this in turn has resulted in sleep disturbances, poor physical health, greater depressive symptoms, high rates of mortality, stroke risk and psychological distress in bereavement [17,44-47]. Given the diversity and complexity of carers and the multiple care circumstances, it may be more relevant to identify those carers at risk of, or experiencing high anxiety levels or poor health, for example, and provide them with support [48]. Extensive research conducted by Nolan and colleagues [49] and more recent research conducted by Montgomery and colleagues [50], suggest individual assessment of a carer’s unique caregiving situation is necessary. Frailty intervention protocols that include individual assessment of a carer’s caregiving situation may help identify those at risk, potentially delivering a more positive and supportive intervention for all involved.

Co-resident carers in this study had statistically significant higher self-esteem than those carers who did not co-reside with their frail older relative. Melis and colleagues [51] found co-resident carers of older people provided more care and were therefore more responsive to home based interventions. Carers who did not co-reside with the frail older participant in this study were generally employed and/or daughters with families of their own. These non-resident carers may have found some components of the frailty intervention an additional responsibility or commitment of time. Melis and colleagues [51] also found that a comprehensive geriatric assessment of older care recipients resulted in non-resident carers being confronted with the reality of the older person’s ‘vulnerability and impairments’. Identification and consideration of non-resident carers in frailty interventions is therefore also recommended.

Carers are often relied upon to encourage adherence to frailty intervention strategies and the responsibility placed on carers during interventional research can make caregiving a complex experience [4]. The results of this study indicate that there is a need for health care professionals to understand the prevalence of anxiety experienced by carers of frail older people. However, the frailty intervention did not have a significant impact on the carers’ schedule or usual activities. Given this finding, the inclusion of carers in interventions targeting frailty may be practical.

### Limitations

The results of this study are limited to those carers providing care to older people who live independently in an urban community. The inclusion of a CONSORT Statement allowed for a transparent reporting process, however, it should be noted that this study had a high rate of loss to follow-up. Forty nine percent (49%) withdrew as a result of the study protocol criteria; either the care recipient was hospitalised, entered a residential aged care facility or died. A substantial number of carers declined follow-up for unknown reasons, this loss of carers to the study may be because these carers did not receive appropriate support or their caregiving became too overwhelming. Whilst the participation rate was higher in the control group at baseline, total loss to follow-up was higher in the control group, particularly at the 12 month measure and the 3 month post frailty intervention measure. Carers are more likely to engage in interventions that offer services that may benefit the care recipient [52], or if their involvement may possibly delay or prevent placement in a residential aged care facility [53]. The carers of the participants receiving the frailty intervention may have been more motivated to participate in the carer study, knowing that their family member was involved in an intervention that aimed to improve their functional ability and general wellbeing.

## Conclusion

Interventions targeting frailty have the potential to positively impact on carers’ health and self-esteem. An individual assessment of the caregiving situation may help identify carers at- risk of physical and mental ill health, and facilitate the provision of information and support that will assist them to continue caring for their frail older family member or friend. Further research that explores the features of frailty interventions that impact on the caregiving experience is recommended.

## Abbreviations

CRA: Caregiver Reaction Assessment; FIT: Frailty Intervention Trial; HADS: The Hospital Anxiety Depression Scale; LMM: Linear Mixed Models Procedure; MAR: Missing at random.

## Competing interests

The authors declare that they have no competing interests.

## Authors’ contributions

CA conceived of the study, participated in its design and carried out the field work, data collection and analysis, and was the main contributor to the manuscript. IC and SR participated in the design of the study, helped to draft the manuscript and provided invaluable guidance. All authors read and approved the final manuscript.

## Funding

This work was supported by a National Health & Medical Research Grant (402791); and a Royal College of Nursing Australia Research Grant. These funding bodies did not play a part in the research protocol, data analyses, data interpretation, or writing of the report.

## Pre-publication history

The pre-publication history for this paper can be accessed here:

http://www.biomedcentral.com/1471-2318/12/66/prepub
